# Negative-pressure wound therapy in the management of diabetic Charcot foot and ankle wounds

**DOI:** 10.3402/dfa.v4i0.20878

**Published:** 2013-09-23

**Authors:** Crystal L. Ramanujam, John J. Stapleton, Thomas Zgonis

**Affiliations:** 1 University of Texas Health Science Center at San Antonio, San Antonio, TX, USA; 2 VSAS Orthopaedics, Allentown, PA, USA

**Keywords:** negative-pressure wound therapy, Charcot foot, diabetes mellitus, neuropathy, plastic surgery, external fixation

## Abstract

As the prevalence of diabetes mellitus continues to rise, innovative medical and surgical treatment options have increased dramatically to address diabetic-related foot and ankle complications. Among the most challenging clinical case scenarios is Charcot neuroarthropathy associated with soft tissue loss and/or osteomyelitis. In this review article, the authors present a review of the most common utilizations of negative-pressure wound therapy as an adjunctive therapy or combined with plastic surgery as it relates to the surgical management of diabetic Charcot foot and ankle wounds.

Negative-pressure wound therapy (NPWT), also known as topical negative-pressure therapy or vacuum-assisted closure, has revolutionized the world of wound care. Morykwas et al. first published on this modality using an open-cell foam dressing with application of a controlled subatmospheric pressure for treatment of acute and chronic wounds in 1997 (1–3). Since then multiple studies have demonstrated the usefulness of NPWT in diabetic foot wounds (4–6). In the diabetic patient, the combination of Charcot neuroarthropathy (CN) and an open wound with or without osteomyelitis further challenges treatment protocols since each of these clinical manifestations carries with it an increased risk for infection and amputation (7). Expedited wound closure is a priority and may decrease morbidity and mortality rates in this population (8). Although NPWT's exact mechanism of action has yet to be elucidated, its success as an adjunct for wound healing has well been established in the surgeon's armamentarium.

Much of the existing evidence supports that the success of NPWT in wound healing is likely multifactorial. A study by Zöch on 10 patients found an increase in blood flow to diabetic foot wounds treated with NPWT measured by IC-view perfusography (9). Kamolz et al. showed a clinically apparent reduction in edema with NPWT used on burn wounds in the hand as a result of fluid removal by the device (10). Even in the early studies by Morykwas et al. using an animal model, he noted an increase in the formation of granulation tissue at wounds through NPWT (2). In a randomized controlled trial of 30 patients with diabetic foot ulcers, Kopp et al. demonstrated an increase of growth factors within the wound fluid of those treated with NPWT compared to the control group treated with a hydrocolloid (11).

As the conventional option for care of diabetic foot wounds has been moist wound dressings, randomized controlled trials comparing NPWT to moist wound healing have populated the literature. Eginton et al. compared the rates of wound healing between NPWT and moist dressings, clearly showing improved healing rates in the diabetic foot wounds treated with NPWT (5). In regard to quicker achievement of wound closure and formation of granulation tissue, a study by Blume et al. also found NPWT superior to standard wound dressings (12). Although this was a randomized controlled trial, the results described by the study should be considered carefully since industry-sponsored research can be biased in reporting mostly positive results when compared to non-industry-sponsored studies. A study by Paola et al. further demonstrated that NPWT reduced the need for subsequent amputations at a 6-month follow-up period when compared to the control group (13). These studies also suggested no differences between the treatment-related complications of NPWT and conventional moist wound dressings (14). Studies investigating the role of NPWT on bacterial clearance and infection control showed inconclusive results (15–17). Furthermore, questions still remain regarding the optimal values for duration of NPWT use and levels of pressure, as well as specific materials used, and their discussion is beyond the scope of this article.

## Clinical case scenarios

Neuropathic wounds in the diabetic CN foot and ankle can manifest through a variety of different pathways and mechanisms. Most common clinical case scenarios that can lead into a CN deformity from a pre-existing neuropathic ulceration(s), osteomyelitis and/or surgical intervention may include but are not limited to the following two clinical presentations: (1) diabetic neuropathic submetatarsal ulcerations that are being treated conservatively with off-loading can potentially alter the biomechanics of the foot and increase trauma to adjacent joints that could initiate the CN process and (2) diabetic neuropathic partial pedal (e.g. toe, partial ray, transmetatarsal) amputations with a recent history of surgical intervention that are permitted to an early weight-bearing status. In the above clinical presentations, the diabetic patient presents with a history of a pre-existing ulceration and/or infection that may have contributed to the development of CN in a different joint(s) with or without a new ulceration. However, it is not yet known whether the incidence of CN is caused by changes in biomechanical function following partial foot amputation, elicited by surgery itself, or triggered by osteomyelitis (18–20).

For any other patient with diabetic CN, identification of the stage of CN is important in formulating a treatment strategy. In the acute stage of CN, inflammation may produce severe edema that when combined with ambulation in the insensate patient can cause significant skin breakdown and/or compromise a pre-existing wound. NPWT can be carefully applied during this stage if a wound is present but must be combined with a period of immobilization in order to halt the inflammatory process, limit peri-wound edema, and maintain the foot and/or ankle architecture to prevent further deformity (21). Immobilization can be in the form of removable splints, casts, prefabricated braces, and external fixation devices that are designed to allow for appropriate access to the wound for regular NPWT dressing changes, while enforcing a non-weight-bearing status through the utilization of a gait assistive device(s) or wheelchair.

As CN can compromise the osseous architecture and stability of the foot and ankle, the chronic form of the condition typically presents with progressive deformity resulting from a cumulative effect of repeated injury that goes undetected or neglected during the acute process. Failure to properly accommodate and off-load braceable deformities or to recognize unstable and/or unbraceable deformities which require surgical intervention may potentially lead to an ulceration and/or infection. In this clinical scenario, patients often present to a medical facility because they have noticed a wound and possibly a misshapen foot, yet cannot recall any of the signs or symptoms of acute CN. For these patients in the absence of infection, the foot and ankle specialist should identify whether the deformity is stable or unstable in order to determine whether surgical intervention may be indicated for correction of an unstable deformity. In a wound with stable chronic CN, sharp debridement of the wound and use of NPWT can facilitate secondary healing, while re-ulceration can be avoided through accommodative shoe gear and/or bracing combined with frequent clinical examinations.

**Fig. 1:**
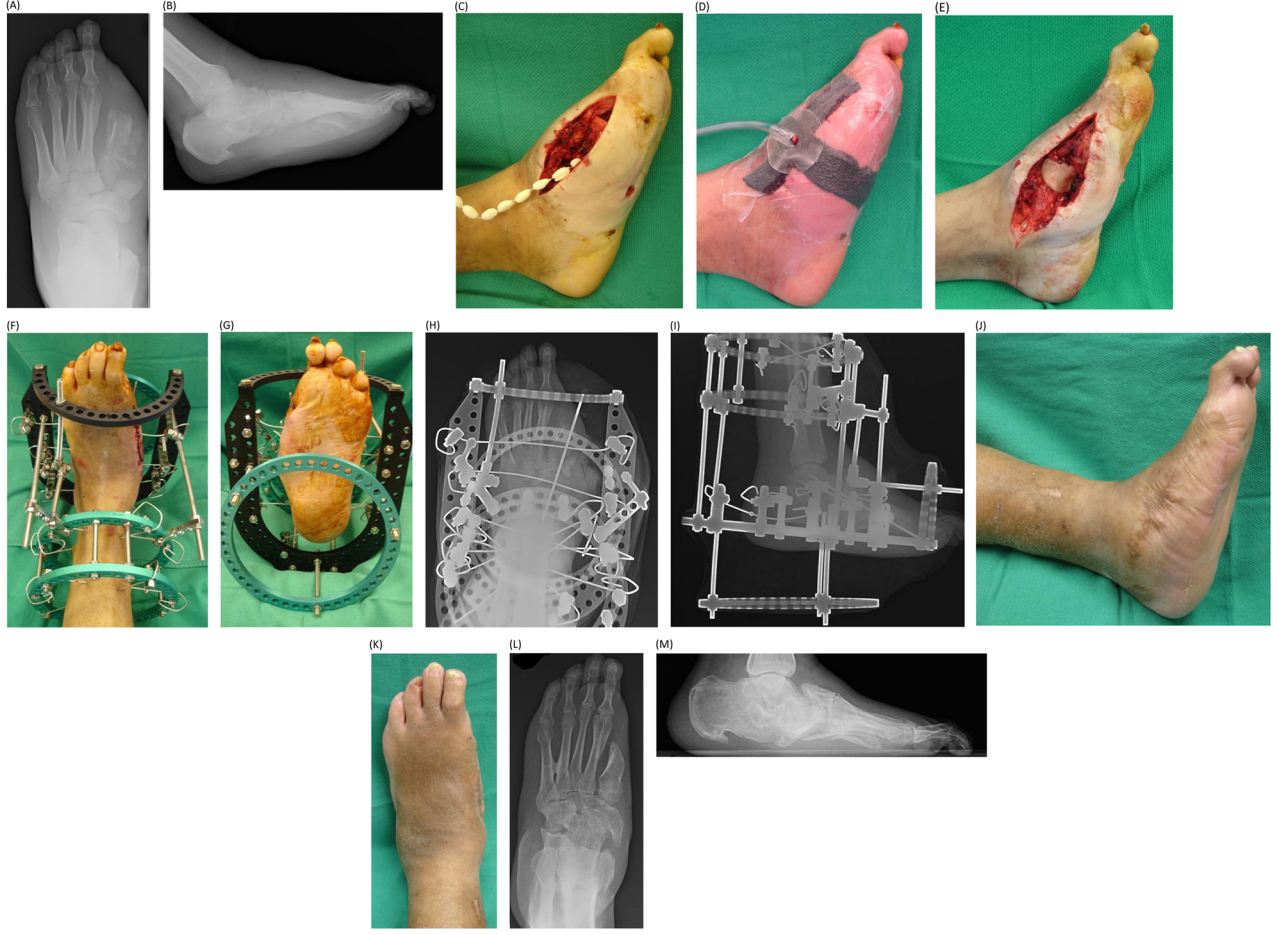
Pre-operative anteroposterior (A) and lateral (B) radiographic views showing the initial presentation of a diabetic Charcot foot with abscess and osteomyelitis. Patient underwent an initial surgical debridement and bone biopsy that was followed by revisional osseous and soft tissue debridement, application of non-biodegradable cemented antibiotic beads and negative-pressure wound therapy 2 days after the initial operation (C, D). The negative-pressure wound therapy was discontinued on post-operative day 6 followed by local wound care dressing changes. At approximately 10 weeks, the patient underwent removal of the non-biodegradable cemented antibiotic beads that was followed by a medial column arthrodesis, allogenic bone grafting and application of a circular, external fixation device for compression and surgical off-loading (E–I). The external fixator was removed at approximately 6 weeks that was followed by local wound care, further lower extremity casting, immobilization, and weight-bearing status with accommodative bracing. Final clinical (J, K) and radiographic (L, M) views at approximately 23 months follow-up.

Delayed or ineffective treatment of CN foot and ankle wounds in the diabetic population can potentially lead to soft tissue infection and osteomyelitis. Appropriate surgical debridement of infected soft tissue and bone often leaves large defects that may be amenable to treatment with non-biodegradable cemented antibiotic beads/spacers. In these surgical case scenarios, NPWT may only be considered for surgical wounds with eradicated bone or soft tissue infection, possess adequate perfusion, and are in need for frequent dressing changes. For exposed tendon structures, a non-adherent dressing can be applied first directly over the tendon prior to placement of the NPWT foam. Furthermore, commercially available open-cell foams of different compositions (white foam or silver-impregnated foam) are designed for placement over bone or in tunneling wounds; however, the efficacy of these materials is yet to be determined. Although NPWT is beneficial as an adjunct of treating these complex case scenarios, a thorough surgical debridement and appropriate antibiotic therapy are necessary to a successful wound healing in the diabetic CN foot or ankle (Fig. 1).

NPWT is also indicated as an adjunct to surgical wound closure utilizing plastic surgical techniques and osseous reconstruction for the unstable and/or unbraceable CN deformity. Due to the effects of NPWT mentioned earlier, this modality can be useful in wound bed preparation prior to definitive wound closure techniques such as skin grafts or flaps. NPWT can also be considered to promote wound healing at flap donor sites. When used cautiously, observed frequently and typically at lower pressure levels, NPWT can also be beneficial directly over skin grafts or flaps during early incorporation. In a clinical trial comparing NPWT with standard non-adherent gauze dressings for skin-grafted diabetic foot wounds, Paola et al. found that the percentage of subjects with complete skin graft healing was increased in the NPWT group (13). Reduction of flap edema, increase in blood flow to the flap, and bolstering of the flap to the recipient wound are all effects which support the use of NPWT in this setting (22). Similarly, rather than using standard bolster-type dressings, NPWT can also be used in combination with orthobiologics for dermal replacement. This technique maintains contact between the biological layer and the wound, reducing micro-motion while also preventing excessive fluid accumulation. In cases of surgical wound dehiscence or partial flap necrosis, NPWT can also be used to facilitate delayed primary closure or healing by secondary intention after adequate debridement.

Similarly, NPWT has also found a role in stabilizing closed incisions which is useful in extensive CN foot and ankle reconstructions where there is a high risk for wound dehiscence. Arthrodesis procedures often used for reconstruction of unstable CN foot or ankle fracture dislocations can produce tenuous incisions and may lead to significant post-operative edema. A study by Kilpadi et al. demonstrated that the use of NPWT over closed incisions can reduce and normalize tissue stresses and bolster appositional forces at the incision site (23). In CN reconstructive cases where primary wound closure is not feasible, NPWT systems can be used in conjunction with off-loading external fixation to encourage wound healing. External fixation provides for proper stabilization of the corrected deformity while simultaneously allowing regular access to the wound(s) for continued care. For diabetic CN patients with chronic foot and ankle wounds who are not optimal candidates for reconstructive procedures, NPWT can stabilize wounds until their comorbidities are controlled and definitive wound closure with corrective osseous procedures can then be safely accomplished.

Prior to application of NPWT in any patient, one should consider the existing contraindications to this modality (24). Absolute contraindications include but are not limited to untreated osteomyelitis or sepsis within the area of the wound, inadequately debrided wounds or those with necrotic tissue and/or eschar, presence of untreated coagulopathies, malignancy at the wound site, and allergy to any materials related to the device. Relative contraindications to NPWT include but are not limited to wounds with active bleeding, patients with poor nutritional status, and patients on active anticoagulant therapy.

## Conclusion

Since its initial development, NPWT has gained widespread acceptance for a broad range of wound indications, including those found among diabetic CN. NPWT can be used to treat CN wounds produced as a result of neuropathy and deformity, following debridement of infection or amputation, and in reconstructive soft tissue and osseous procedures. Several studies emphasize that treatment outcomes are often based on the specific techniques and materials used for NPWT application, therefore additional suitably powered, high-quality clinical trials are needed to fully determine efficacy. Careful patient and procedure selection along with appropriate technique is imperative for successful use of NPWT in the diabetic CN foot and ankle.
